# Cognitive Impairment in Hospitalized Seniors

**DOI:** 10.3390/geriatrics1010004

**Published:** 2016-01-05

**Authors:** Monidipa Dasgupta

**Affiliations:** Division of Geriatric Medicine, Western University, University Hospital, 339 Windermere, London, ON N6A 5A5, Canada; monidipa.dasgupta@sjhc.london.on.ca; Tel.: 1-519-685-8500 (ext. 33922)

**Keywords:** Cognition disorders, hospitalisation, delirium, dementia

## Abstract

Cognitive disorders are highly prevalent in hospitalized seniors, and can be due to delirium, dementia, as well as other disorders. Hospitalization can have adverse cognitive effects, and cognitive dysfunction adversely affects hospital outcomes. In this article, the literature is reviewed on how hospitalization affects cognitive function and how cognitive impairment affects hospital outcomes. Possible interventions in cognitively impaired hospitalized seniors are reviewed.

## 1. Introduction

Cognitive impairment is prevalent in acute care hospitals and can be related to various conditions. Delirium is common in hospitalized seniors occurring in 11%–42% of medical in-patients [1]. Dementia is also common; a systematic review based on 14 studies found that dementia (diagnosed with validated criteria) was present in 12.9%–63% of medical or non-hip fracture surgical patients. Variations in rates were felt to be related to different study populations with differences in important confounders, such as age [2]. Dementia and delirium frequently co-exist, and dementia is a very strong risk factor for delirium. Sub-syndromal delirium, in which affected individuals meet some but not all of the diagnostic criteria for delirium, is another cause of cognitive impairment in hospital [3,4,5]. In one systematic review, pooled estimates of prevalence and incidence rates of sub-syndromal delirium were 23% and 13%, respectively [6]. Reversible cognitive decline not otherwise specified (defined by an improvement in Mini-mental status exam (MMSE) scores during hospital stay) is another cause for cognitive impairment in hospitalized seniors [7,8,9].

The high prevalence of cognitive disorders in hospitalized seniors is likely a reflection of multiple factors. Cognitive impairment is prevalent in seniors, and in some medical conditions that often result in hospitalization. For instance, the reported prevalence rates of cognitive impairment in congestive heart failure, Chronic Obstructive Pulmonary Disease (COPD), and Diabetes Mellitus vary between 25% and 74% [10,11,12]. Multi-morbidity is prevalent in seniors, and isolated disease states are less common [13,14,15]. In addition, hospital factors may also contribute to new cognitive disorders, such as delirium.

Given the high prevalence of cognitive impairment in hospitalized seniors, hospital-based clinicians will encounter this patient population frequently. Although reviews on specific cognitive disorders, such as delirium, have been published, there are no reviews of general cognitive disorders in hospital. The purpose of this clinical review is to provide a general overview of what studies reveal about the inter-relationship between hospitalization and cognitive impairment (not otherwise specified), and the associated implications. Possible interventions are also discussed.

## 2. Methods

Prior systematic reviews have focused on specific causes (or management approaches) of cognitive impairment, such as delirium or dementia. However, the topic for this review is cognitive impairment not otherwise specified, and is broader and not limited to a single question or diagnostic category; therefore, a systematic review was not undertaken. However, a search strategy was conducted using Medline and the MeSH terms “cognition disorders” OR “delirium” OR “dementia” AND combining this with “hospitalization”. The search was limited to human studies, conducted in English, and restricted to adults 65 years or older; studies pertinent to the inter-relationship between hospitalization and cognitive disorders were included. Studies focusing on psychiatric in-patients or alcohol-related cognitive disorders and pharmacologic management of cognitive/behavioral disorders were excluded. Reference lists of studies were also searched for relevant articles. No specific methodological inclusion or exclusion criteria were pre-specified, but in general, for prognostic studies, prospective studies (with sample sizes of at least 200 participants) are presented when available (or very large database studies). For intervention studies, articles with concurrent control groups are presented preferentially if available; otherwise articles without control groups are presented (in intervention studies that reveal relevant preliminary results).

## 3. Results of Review

### 3.1. The Effect of Hospitalization and Co-Morbidity on Cognition

At least six prospective studies have linked hospitalization to future cognitive decline (see Table 1) [16,17,18,19,20,21]. In these studies, community dwelling adults were followed longitudinally, and cognitive status was measured prospectively. Rates of cognitive decline prior to and after hospitalization were calculated, and changes in rates of decline were compared to individuals without intervening hospitalization. Hospitalization was generally associated with a subsequent acceleration in the rate of cognitive decline. In one study [16], hospitalization resulted in a 2.4-fold increase in the rate of cognitive decline compared to the rate prior to hospitalization. Cognitive decline after hospitalization was more marked in those demonstrating some cognitive decline prior to hospitalization, thus suggesting hospitalization may hasten the rate of cognitive decline in cognitively declining individuals. However, hospital-related cognitive decline has also been demonstrated in individuals without cognitive impairment, and some studies suggest that hospitalization is associated with a higher rate of incident dementia [17,18]. In community-dwelling seniors with Alzheimer’s dementia, hospitalization with delirium was associated with an increased rate of cognitive decline [19]. A greater than two-fold increased rate of cognitive decline has been observed in survivors of hospitalization for diverse diagnoses including pneumonia, myocardial infarction, or strokes [18,20,21].

There are likely multiple ways by which hospitalization impacts cognition. Hospitalization adversely affects functional abilities in seniors, with about one third of seniors losing functional abilities after hospitalization [22,23,24]. The same hospital processes that affect functional abilities [25] may impact cognition. For example, immobility, which contributes to functional decline after hospitalization, has also been associated with delirium. Improving mobility has been shown to shorten delirium duration [26] and helps to prevent delirium [27]. Other hospital processes linked to delirium symptoms include lack of sensory aids, room changes, disorientation, the use of Foley catheters, physical restraints, use of at least three new drugs, and lack of family members [28,29]. Contact precautions have also been associated with worse psychological outcomes [30]. Although it may be difficult to sort out how much of the decline in cognition and function is related to hospital processes (*versus* the acute medical condition(s) and underlying frailty of the hospitalized individual), delirium intervention studies that target hospital processes can decrease incident delirium [31] by one third. Another study demonstrated a lower rate of delirium in individuals randomized to receive rehabilitation at home *versus* routine in-patient rehabilitation [32]. This would suggest that hospital processes may partially be responsible for the cognitive and functional decline after hospital stay, beyond the effects of the acute illness(es) themselves.

**Table 1:** Prospective cohort studies monitoring cognitive function before and after hospitalization.

Study Authors	Sample Characteristics	Follow-up (Time, in Years)	Cognitive Measures	Findings
Wilson *et al.*, 2012 [16]	Community-dwelling seniors (*n* = 1870)	9 years	Four cognitive tests (immediate and delayed recall of East Boston story, oral versions of symbol digits test, MMSE)	Increased rate of cognitive decline after all-cause hospitalization (from Medicare records) (*p* < 0.001)
Ehlenbach *et al.*, 2010 [17]	Community-dwelling, non-demented seniors, from HMO (Seattle area) (*n* = 2929)	6.1 years	Screening with Cognitive abilities screening instrument (CASI); further clinical assessment for dementia if indicated	Faster rate of decline after all-cause (excluding brain injury) hospitalization; higher rate (*p* = 0.001) of incident dementia (DSM criteria)
Tate *et al.*, 2014 [18]	3069 community-dwelling seniors without dementia, enrolled in a RCT	6.1 years (median)	Teng’s modified Mini-mental status (3MSE), Alzheimer’s Disease assessment scale, Clinical dementia rating scale; refer for further neuropsychiatric tests, expert panel for dementia diagnosis	Faster rate to dementia diagnosis after pneumonia hospitalization (*p* < 0.0001) and after hospitalization with other infections (*p* < 0.0001)
Fong *et al.*, 2012 [19]	Community-dwelling seniors with Alzheimer’s dementia (*n* = 771) (part of Massachusetts Alzheimer’s research center)	2 years (median)	Information memory concentration subset of Blessed memory dementia scale, Dementia Severity scale, Clinical assessment	Higher rate of cognitive decline (RR 1.6, 95% CI 1.2–2.2) if hospitalized generally (all causes) with concurrent delirium
Davydow *et al.*, 2013 [20]	Nation-wide (USA), prospective longitudinal study of individuals 50 years or over, (*n* = 1434), surviving hospitalization for pneumonia, MI, or CVA (subset of Health and Retirement Study)	Followed for 7.7–9.8 years before and 9.9–12.7 years after hospitalization	Telephone Interview for Cognitive Status (TICS)	Faster rate of moderate–severe cognitive impairment after hospital with diagnoses of pneumonia, myocardial infarction, or stroke (no other diagnoses considered) (*p* = 0.03)
Shah *et al.* 2013 [21]	Participants of cardiovascular Health Study (high functioning community-dwelling seniors, from 4 American states) (*n* = 5888)	Followed over 10 years	Teng modified Mini-mental status state (screening); subgroup underwent further neuropsychological testing if indicated	Increased hazard ratio (*p* = 0.01) for dementia after pneumonia hospitalization (but not other infections; patients were included if they had pneumonia, severe pneumonia, sepsis, or other infectious causes for hospitalization)

The hospital environment may also be difficult for chronically cognitively impaired individuals. Patient-centered care is recommended for individuals with dementia, but there are multiple barriers to providing patient-centered care in the acute environment [33,34]. These include space limitations (which can limit mobility), a focus on the physical over psychological needs of patients, difficulty in providing time-consuming care in a stressful acutely changing environment, disruptions in the environment resulting from room changes, and lack of staff training. In addition, the recognition of cognitive impairment has been reported to be poor by all members of the health care team [35].

Apart from the general effects of hospitalization, certain co-morbid conditions are associated with cognitive impairment. Cognitive impairment is very common in hospitalized individuals with congestive heart failure (CHF), with one systematic review citing prevalence rates of 25%–74% [10]. Severe cognitive impairment has been associated with increasing severity of heart failure (based on NYHA class) symptoms in a study enrolling 611 people with CHF [36]. The specific mechanisms underlying this association still have to be elucidated, but some mechanisms have been suggested. A cross-sectional study of 57 individuals with heart failure and no history of dementia found that lower cognitive scores correlated with low ejection fraction (≤30%) [37]. CHF with concurrent hypotension (defined as systolic blood pressure < 130 mm Hg) has been associated with lower cognitive functioning in a study of 1583 individuals hospitalized with CHF [38]. These studies could suggest that hypoperfusion may partially underlie the cognitive dysfunction observed in CHF. In an observational study of 1220 patients hospitalized with CHF, treatment with Angiotensin-Converting Enzyme Inhibitors was associated with an improvement in cognitive functioning on discharge [39]. Although not a randomized control trial, this suggests that optimizing treatment of an underlying medical condition may improve cognitive functioning.

In another observational study, 66 delirious individuals who had electrolyte disturbances and were referred to a psychogeriatric consultation liaison service were followed. In this study, delirium symptoms improved faster if electrolyte disorders were treated. Although not a randomized control trial, this also would suggest that optimizing an individual’s medical status may improve cognition [40].

Other prospective, large, community-based studies have shown hypertension [41,42] or hypotension [43], diabetes mellitus [42,44,45], and severe COPD with hypoxia [46,47] to be associated with future worsening cognitive impairment. This would suggest that these co-morbid conditions may further contribute to cognitive impairment through various pathophysiologic processes; for example, in diabetes mellitus, the brain may itself be affected directly by insulin resistance [48]. These co-morbidities may also partially contribute to cognitive decline after hospitalization.

### 3.2. The Effect of Cognitive Impairment on Hospital/Disease Outcomes

Just as hospitalization or co-morbidities can adversely affect cognitive function, cognitive dysfunction also affects hospital outcomes. It is well-established that delirious patients do poorly both in the short-term and the long-term (increased length of stay, institutionalization rates, mortality, and risk for dementia), regardless of the underlying illness or other important confounders [1,49,50,51,52]. Death rates at hospital discharge after delirium have ranged between 14.5% and 37% [1]. Delirium is a negative prognostic indicator, regardless of the underlying illness, and should therefore be taken seriously by all care providers.

Cognitive impairment, not restricted to delirium, has also been variably associated with adverse outcomes. In a systematic review of dementia in hospitalized adults, increased length of stay and functional decline was associated with dementia, but this was based on very few studies and authors were cautious about making broad conclusions, given the frequent confounding factors that can accompany dementia (e.g., advanced age, malnutrition, *etc*.) [2]. Studies do support a higher prevalence of malnutrition and functional limitations on admission to hospital in cognitively impaired seniors [53,54], as well as increased falls risk [55,56]. These may impact mortality or other adverse hospital outcomes.

The findings of studies assessing the association of dementia with in-hospital or short-term (up to 1 year post discharge) mortality are variable (Table 2). While some cohort studies in which cognition is measured prospectively [35,57,58,59,60,61], or large database studies where variables are collected from medical records [13,62,63,64,65], suggest increased short-term mortality with dementia or cognitive impairment, this has not been confirmed by all studies [66,67]. Some studies suggest that other variables may explain some of the increase in mortality observed in cognitively impaired individuals, such as co-morbidity, malnutrition [66], increased adverse event rates [56], and underlying frailty [68]. One study suggested a differential effect of dementia on mortality depending on the hospitalized individual’s age (specifically the increase in mortality may be more marked in younger individuals with dementia [64]). Some studies have suggested that increasing severity of cognitive impairment correlates with a graded increase in subsequent mortality [57,58,60].

Other ways in which cognitive impairment can affect hospitalization is by the presence of cognitive and behavioral symptoms, which may result in increased lengths of stay [69,70]. The burden of behavioral symptoms ranges from 27% to 54% in studies of dementia or delirium [69,71]. These can include retarded speech or movement, suspiciousness, aggression, refusing treatments, and can result in restraint, sitter, or security use. Providing care for individuals with behavioral symptoms can therefore be challenging. Some studies have also suggested that care practices may differ in individuals with cognitive impairment. There are differences in consult services requested as well as testing and procedures performed [72]. Inappropriate prescribing practices may also be more prevalent in cognitively impaired individuals as found in one study that used a French adaptation of the STOPP/START criteria to judge inappropriate prescribing [73]. Another study reported that adverse effects were also associated with cognitive impairment, which was frequently undiagnosed [56].

Cognitive impairment can also affect subsequent management of chronic diseases. For instance, in a retrospective analysis of administrative data involving 15,996 older recipients of drug-eluting stents, a diagnosis of dementia was associated with not filling a prescription for clopidrogel within 7 or 90 days of discharge (failure to fill a clopidrogel prescription was also associated with increased mortality) [74]. In a multi-site, case-control study on hospitalized individuals in Denmark, individuals exhibiting cognitive impairment (as documented in the medical record) were more likely to have hospital admissions that were judged to be related to preventable adverse drug reactions [75].

In a multi-center study of 744 hospitalized CHF patients, increasing severity of cognitive impairment was associated with an inability to recognize symptoms of poor health or worsening CHF [76]. Cognitive dysfunction that was not recognized in a prospective study *(n =* 282) of seniors hospitalized with CHF who were not delirious and not fully dependent for their ADLs was associated with increased re-hospitalization or death rates within 6 months of discharge from hospital [60]. Executive dysfunction, in community-based studies of diabetics, has also been associated with increased health care service utilization, difficulty in managing diabetic medications, and dietary lifestyle changes [77]. Cognitive impairment has been associated with increased risk of hypoglycemic events requiring medical attention [78]. Smoking cessation is more difficult to achieve for cognitively impaired individuals [79]. It is likely that cognitive impairment affects disease management after hospital discharge as well.

**Table 2:** Studies assessing the association between cognitive impairment (or dementia) and death (studies either prospectively measured cognitive function or used large databases and medical records). COPD: Chronic Obstructive Pulmonary Disease; CHF: Congestive Heart Failure; LOS: length of stay.

Study Authors	Sample Characteristics	Type of Study	Confounders Assessed	Effect of Cognitive Impairment on Mortality
Torrison *et al.*, 2012 [35]	200 patients, ≥60 years of age, admitted to general medical units	Prospective study	Adjusted for gender, home care use, co-morbidity	Increased 12 month mortality if cognitively impaired
Marengoni *et al.*, 2013 [57]	66 centers in Italy, ≥65 years, admitted to internal/geriatric wards (*n* = 1201)	Prospective study	Controlled for age, gender, education, other diseases, comorbidity, functional status, adverse events	Increased in-hospital mortality in cognitively impaired individuals who had an adverse event
Sampson *et al.*, 2009 [58]	617 patients, ≥70 years old, admitted to hospital, excluded if persistently delirious	Prospective study	Controlled for age, Acute physiology disturbance score, LOS, co-morbidity, and Waterlow scale (measure of frailty)	Increased in-hospital mortality, largely explained by confounders including frailty measure
Vetrano *et al.*, 2014 [59]	1123 hospitalized seniors, ≥65 years of age (7 Italian hospitals)	Prospective data gathering		Primary outcome was length of stay. However, in-hospital mortality was increased in dementia
Dodson *et al.*, 2013 [60]	282 hospitalized for CHF, ≥65 years of age (excluded delirious, severe functional dependent individuals, and if right-sided heart failure)	Prospective study	Adjusted for age, race, kidney disease, and use of aldosterone receptor antagonist	Death or re-hospitalization at 6 months more likely in severely cognitively impaired
Zuccala *et al.*, 2003 [61]	1113 patients admitted for heart failure in 81 hospitals (Italy)	Prospective study	Age, gender, certain co-morbid diagnoses, cardiovascular medications	Increased in-hospital mortality (and 1-year mortality )
Chaudhry *et al.*, 2010 [13]	62,330 persons hospitalized with CHF	Medical record-based (database)	Adjusted for weight, CHF medication use, pulse pressure	Increased 30-day post-discharge mortality
Banta *et al.*, 2010 [62]	15,497 patients hospitalized with CHF at a VA center	Medical record-based		Increased in hospital mortality
Guijarro *et al.*, 2010 [63]	More than 3.3 million people discharged from 32 hospitals	Medical record-based	Controlled for other diagnoses	Increased higher in-hospital mortality
Draper *et al.*, 2011 [64]	253,000 hospitalized individuals 50 years of age or older	Medical record-based	Age	Increased mortality rate, particularly for younger demented individuals
Liao *et al.*, 2015 [65]	Hospitalized patients with COPD (excluded mental illnesses, apart from dementia) (*n* = 6740)	Medical record-based	Controlled for age, gender, admission year, co-morbidity, infection site, hospital level and LOS (cases with dementia, controls without)	Increased in-hospital mortality
Li *et al.*, 2013 [67]	Hospitalized patients (*n* = 34,888), excluding obstetric, psychiatric or brain injury diagnoses	Medical record-based		No increase in mortality at discharge

### 3.3. Possible Interventions for Cognitive Impairment in Hospitalized Seniors

As already discussed, delirium prevention is effective in about one third of cases [31,80]. Well-studied programs such as the Hospital Elder Life Program (HELP), mediated through hospital volunteers, are effective at preventing delirium [27], and have been adapted in various hospitals world-wide [81,82,83]. The HELP program involves actively intervening in hospital factors associated with incident delirium, including immobility, disorientation, limiting the use of psychotropic drugs, pro-actively monitoring for dehydration, and provision of sensory aids.

Other methods of proactively intervening on delirium risk factors have been studied and found to be effective. For instance, in a randomized controlled trial (RCT) of older medical in-patients, family members were asked to assist in non-pharmacologic delirium prevention interventions (focusing on provision of sensory aids, extended visiting hours, having familiar objects, and re-orientation techniques) and the intervention resulted in a 59% reduction in incident delirium [84]. In another study, community-dwelling seniors admitted to a non-teaching service were assigned to a control team or an intervention comprising an inter-disciplinary team. Members of the inter-disciplinary team were trained to perform daily comprehensive geriatric assessments, and this resulted in decreased delirium rates [85].

In the surgical (hip surgery) setting, placebo controlled trials have shown that active prevention is effective in decreasing delirium. These studies used comprehensive geriatric assessments and assessed for and intervened on delirium risk factors, and used either ward-based models [86], or active consultative models [87,88,89]. However, one recent RCT in which comprehensive geriatric assessment was done in an orthogeriatric ward, did not result in decreased delirium in hip fracture patients, although fewer individuals were discharged with delirium in the intervention group [90]. In this latter study, close to 50% of enrolled patients had dementia at baseline.

Unfortunately, once delirium has occurred, less is known about how to improve outcomes. Although some studies suggest that ward-based, multifaceted, non-pharmacological interventions may shorten the duration of delirium or adverse effects [91], this has not been shown consistently in other studies [92,93]. Much remains unknown about how to improve outcomes when an individual is actively delirious. Observational, preliminary studies suggest a possible benefit of specialized delirium wards or rooms [94,95], or of bright light therapy [96,97] in decreasing falls, restraint use, or delirium symptoms, but these studies are small, preliminary observations, and firm conclusions cannot be made about the effectiveness of these interventions. Delirious individuals are at high risk for complications and re-admission [5,71], and one before–after study has suggested a possible benefit from having a case manager and rehabilitation intervention after discharge in delirious individuals [98].

There is also a paucity of intervention studies directed at hospitalized cognitively impaired individuals, with dementia or other causes of cognitive impairment. One recent intervention study enrolled 202 hospitalized seniors over the age of 65 with general cognitive impairment (detected through active screening) to one of three interventions (assignment was based on which hospital individuals were admitted to). The active intervention involved monitoring and documenting cognitive impairment, with follow-up throughout hospitalization and a transitional care model after discharge home (which included telephone calls and home visits). The intervention resulted in a lower re-hospitalization rate [99]. Another recent study suggested that there may be improved patient and caregiver satisfaction with the use of a specialized medical and mental health unit in individuals with both dementia and delirium. However, there were no differences in use of institutional days [100]. Special care units within the acute care setting, in which a philosophy of unlimited safe ambulation and emphasis on therapeutic activities in a more home-like environment run by specially trained staff have been adapted in some centers, but hard outcome data comparing them to a concurrent control group are lacking [101,102]. Two small studies have suggested a possible beneficial role in decreasing cognitive decline by using cognitive stimulation [103,104]. Studies using computerized alert systems have not been found to be effective in decreasing falls, incident delirium, or other adverse outcomes or practices [105].

### 3.4. Implications of Review Findings

The prevalence of cognitive impairment is high in hospitalized seniors and complicates management of virtually all acute illnesses. Certain diseases are associated with cognitive decline, but processes inherent to hospitalization itself may also contribute to faster cognitive decline. Delirium, in some cases, is preventable, which would suggest that at least one cause of hospital-associated cognitive decline is preventable. Hospital associated processes like immobility, lack of attention to the need for sensory aids, or lack of easy access to hydration are all risk factors associated with delirium. Multiple different models of delirium prevention programs have demonstrated effectiveness in preventing delirium, suggesting that hospitals can and should actively monitor for and try to prevent delirium.

However, there are many gaps in knowledge, including how to best improve outcomes in an actively delirious individual or someone who suffers from cognitive impairment, not otherwise specified. Some cognitive impairment may be related to the underlying active medical issues. Randomized controlled trials looking at the management of medical illnesses should include cognitive outcomes as well, especially in conditions such as CHF where the prevalence of cognitive impairment is high. Cognitive impairment may affect the recognition of symptoms and management of chronic medical illnesses, suggesting that screening for and intervening on cognitive impairment (perhaps by spending more time in educating patients or actively educating caregivers, and having close post-discharge follow-up), may help to better manage disease after hospital discharge.

Other factors to consider when assessing an individual with cognitive impairment are the possible accompanying behaviors. Providing care to these individuals is difficult, and extra support to staff and an interdisciplinary approach may be helpful. Modifying care from a disease-focused to a more patient-centered care approach may be helpful, but training of all staff, including physicians, on how to do this would be required. More research in this area would also help to guide how to best manage behaviors in the acute care environment. Innovative ways of providing care should be studied more, such as bright light therapy or cognitive rehabilitation, specialized units (where behaviors are considered an important part of overall management of the acute illness), and on-going active follow-up after discharge. Intervening on malnutrition and promoting mobility (even when people are at risk for having falls) are other interventions that may help offset the poor outcomes in cognitively impaired hospitalized seniors, and interventions to optimize these factors should be studied.

## 4. Conclusions

Cognitive impairment is very prevalent in acute care environments and can be related to multiple disorders. Hospitalization and certain co-morbid conditions can worsen cognitive function. Similarly, cognitive impairment portends a poorer prognosis in hospitalized seniors. There are likely multiple factors involved in this inter-relationship. Although delirium is potentially preventable, much remains unknown for the treatment and management of cognitive disorders in the hospital environment. Further research is required to improve outcomes of cognitively impaired hospitalized seniors and cognitive outcomes after hospitalization.
